# Retrospective study of lameness in beef cattle in northeastern Sardinia, Italy

**DOI:** 10.1371/journal.pone.0285840

**Published:** 2023-05-23

**Authors:** Sarah Morrone, Antonio Scanu, Masala Gerolamo, Alberto Maria Crovace, Stella Maria Teresa Romeo, Laura Saderi, Giovanni Sotgiu, Nicolò Columbano

**Affiliations:** 1 Department of Veterinary Medicine, University of Sassari, Sassari, Italy; 2 Department of Medicine, Surgery and Pharmacy, Clinical Epidemiology and Medical Statistics Unit, University of Sassari, Sassari, Italy; Universidade Federal de Mato Grosso do Sul, BRAZIL

## Abstract

Lameness is one of the most prevalent diseases affecting dairy and beef cattle, resulting in decreased animal performance, decreased animal welfare, and substantial economic loss. In extensive beef cattle farming, the risk factors for this multifactorial disease are largely unexplored. This study aims to conduct a preliminary epidemiological survey of risk factors in beef cattle in extensive breeding, evaluate the farmer’s perception of lameness, and determine the recurrence frequency of the pathologies under investigation in treated animals. The study was conducted in Sardinia, Italy. The population of the study consisted of 14379 cattle from 230 farms. An ad hoc questionnaire was developed to collect all the necessary data. A strong association was found between breed and the occurrence and recurrence of lameness (p < 0.0001). In addition, the Country of origin of both bulls and cows was found to be correlated with the incidence of lameness (p < 0.0001 and 0.0001, respectively). Farmers who indicated on the questionnaire that lameness was not important on their farm had more animals with recurrences (p < 0.0001) than other farmers. The veterinarian’s treatment choice differed significantly by farmer concern (p = 0.007) and was associated with less disease recurrence (p < 0.0001), resulting in greater farmer satisfaction (p < 0.007). Pure cow breed, French bull origin, and farmer’s age were detected as significant predictors of lameness issues, with pure cow breed and French bull origin having the strongest associations (p = 0.009). Even though the results of this study are preliminary, they indicate that breed selection is crucial in extensive beef farms to reduce lameness prevalence. In addition, it would be reasonable to train breeders to prevent and diagnose lameness early in order to collaborate with veterinarians to prevent recurrence.

## Introduction

Lameness is a painful condition characterised by gait abnormalities and discomfort emerging from the presence of foot or limb lesions [1,2]. Foot problems account for approximately 90% of all lameness cases in dairy cattle, and 70% in feedlots [3]: they lead to significant economic losses following a decrease in milk production, fertility, slaughter weight, and carcass value of affected cows [4,5]. Included in the costs associated with lameness in cattle are the assumption of treatment fees by farmers, revenue losses due to deaths, and the preemptive sale of cattle non-responsive to treatment [6].

Lameness has a multifactorial etiology, and risk factors can be categorised into cow, environmental, management, and nutritional. Cow-related risk factors are: parity (number of calving), breed, age, stage of lactation, and body conformation (e.g., body depth, udder depth, and rear leg side) [7,8]. Environmental and management risk factors include concrete surfaces [9], season, frequency of hoof trimming [10–12], appropriate maintenance of cow tracks, and inappropriate animal handlings [13–15]. Several dietary risk factors were associated with lameness, such as clinical and subclinical ruminal acidosis and feeding on high protein/low fiber lush rye grass pastures and clinical and subclinical ruminal acidosis [16–18].

Extensive cow-calf farming systems are prevalent in the North-Eastern region of Sardinia Island (Gallura, Italy): Farms range from 5 to 150 heads of cattle and pasture is the primary feeding resource. Animals are commonly kept outdoors throughout the year, or at least for extended periods of time, requiring minimal investment in terms of structures and equipment. Animals are typically purchased at about 15 to 18 months of age from fattening centres in Northern Italy, and the presence of one or more bulls on farms ensures reproduction [19].

Most of lameness studies were focused on dairy and beef cattle from intensive production systems in North America and Europe [20]; poor evidence has been published on lameness in extensive production systems. Currently, there is no evidence on factors favouring extensively pasture-raised beef cattle to these diseases.

The purpose of this study was to conduct a preliminary epidemiological survey to assess the incidence of foot pathologies in extensive beef cattle farms at Gallura, Italy, as well as to detect any risk factors. Additionally, it was evaluated farmer’s perception of lameness and treatments performed by veterinarians, podiatrists, and breeders, the recurrence of the pathologies.

## Materials and methods

The study was carried out in extensive beef farms registered in the National Registry Database (BDN Banca Dati Nazionale). Study locations were selected based on the high concentration of beef farmers. A total of 293 beef cattle farms were contacted by phone and email. Farms were contacted randomly and informed on study objectives, selection criteria, and study design. Inclusion criteria were consent of the farmer, healthcare records, and herd size of at least 10 cows. A total of 230 farmers agreed to participate. Farms were visited from December 2021 to March 2022 and the survey evaluated the previous five years (2016–2021).

Farmers were interviewed using an ad hoc questionnaire. Data were collected and analyzed using the website www.evalandgo.it. The survey consisted of 41 items, including single- and multiple-choice, rated, scaled and open-ended response questions (see S1 File).

The first section of the questionnaire was focused on the age of the farm, previous ownership, age of farmers, whether farmers performed alternative professional occupations besides cattle farming, and the number of hours spent on cattle breeding.

The following section included questions on farm management, including farm system carrying out soil remediation, water management, and reproductive and food management. Farmers were asked to clarify if insemination of cows is performed artificially or naturally with bulls, type of feed (exclusive grazing, supplementation with hay and/or feed), and, in case of feed supplementation, its frequency.

The third section described the characteristics of the animals in the farms: total number of cattle; breeds raised; genetic breed purity for both bulls and cows. Additionally, the country of origin of the bulls and cows was requested.

The fourth section described foot diseases. With the support of the International Committee for Animal Recording (ICAR) atlas [21], farmers were asked to detail foot pathologies during the last five years and images were provided to help farmers better classify the clinical conditions. Then, farmers were asked to rate from 0 to 5 their concerns related to foot problems and to report on the weight loss of affected animals and the associated economic losses.

Then, farmers were asked about treatments to affected animals, relapse following therapy, level of satisfaction about treatment. The questionnaire was administered to all breeders and to four expert veterinarians (N.C., A.S., AMC, GM) with a PhD student (S.M.) and a resident (SMTR) visiting the selected farms. Data submitted by farmers on farm management, soil, herd size, number of animals, animal genetics were carefully evaluated.

An electronic form was used to collect all the study variables.

Qualitative and quantitative variables were summarized using relative frequencies (percentages) and medians (interquartile ranges, IQR), respectively. Shapiro-Wilk test was used to verify the normal distribution. Chi-squared or Fisher exact tests were performed to assess statistically significant differences for qualitative variables between the following groups: farms with or without lameness problems, farms with or without recurrences, level of farmer satisfaction with treatment and level of farmer concern with foot disease. Logistic regression analysis was performed to assess the relationship between farm characteristics and lameness issues. A two-tailed p-value <0.05 was considered statistically significant. All the statistical analyses were performed with the statistical software STATA version 17 (StataCorp, College Station, TX, USA).

### Ethics statement

Each recipient of the questionnaire received and signed a written informed consent for the management personal data, based on Italian legislation on privacy. The study protocol was approved by the Ethics Committee of the University of Sassari (approval number 37507).

## Results

A total of 14,379 cattle from 230 farms in 21 municipalities were included. This figure accounts for approximately 91 percent of the total number of cattle in the study area. 141 (61.3%) farmers reported foot disease during the previous five years. 72 (51.1%) reported recurrences. 182 (79.13%) of breeders are involved in farming on a full-time basis, whereas 42 (20.87%) have additional occupations. Most of the farms were inherited.

Median (IQR) age of the farmers was significantly different (p = 0.03) between farms with and without lameness issues (Table 1): 43 (34–51) VS. 48 (38–58) years. Inherited farms were significantly more prevalent (p = 0.001) in farms with (90.1%) VS. those without (73.0%) lameness issues. 71.6% of cows in farms with lameness issues had a French origin in comparison with 53.9% in those without (p = 0.006).

**Table 1:** Farms and farmers characteristics stratified between absence or presence of lameness issues in the farms.

			Total (n = 230)	Farms without lameness issues (n = 89)	Farms with lameness issues (n = 141)	p-value
Median (IQR) farmer’s age, years			44 (35–56)	48 (38–58)	43 (34–51)	0.03
Median (IQR) farm’s age, years			17 (10–30)	21 (10–32)	16 (10–30)	0.63
Education level, n (%)	Elementary school diploma		25 (10.9)	13 (14.6)	12 (8.5)	0.29
	High school diploma		72 (31.3)	32 (36.0)	40 (28.4)	
	Bachelor’s degree		13 (5.7)	5 (5.6)	8 (5.7)	
	Master’s degree		15 (6.5)	3 (3.4)	12 (8.5)	
	Other		3 (1.3)	1 (1.1)	2 (1.4)	
Median (IQR) no. of animals per farm			63 (35–92)	63 (37–95)	63 (35–70)	0.35
No. of animals per farm, n (%)	0–30		31 (13.5)	12 (13.5)	19 (13.5)	0.60
	31–60		46 (20.0)	15 (16.9)	31 (22.0)	
	61–90		90 (39.1)	33 (37.1)	57 (40.4)	
	91–120		56 (24.4)	25 (28.1)	31 (22.0)	
	>120		7 (3.0)	4 (4.5)	3 (2.1)	
Full-time activity, n (%)			182 (79.1)	70 (78.7)	112 (79.4)	0.89
Inherited farm, n (%)			192 (83.5)	65 (73.0)	127 (90.1)	0.001
Recurrences, n (%)			72/141 (51.1)	-	72/141 (51.1)	-
Diet, n (%)		P***	46 (20.0)	19 (21.4)	27 (19.2)	0.91
		P+H***	76 (33.0)	31 (34.8)	45 (31.9)	
		P+H+CC***	94 (40.9)	34 (38.2)	60 (42.6)	
		P+H+CA***	14 (6.1)	5 (5.6)	9 (6.4)	
Soil, n (%)		Rocky (R)	80 (34.8)	35 (39.3)	45 (31.9)	0.39
		Sandy (S)	3 (1.3)	1 (1.2)	2 (1.4)	
		50% R-50% S	81 (35.2)	34 (38.2)	47 (33.3)	
		75% R-25%S	41 (17.8)	12 (13.5)	29 (20.6)	
		75% S-25% R	25 (10.9)	7 (7.9)	18 (12.8)	
Land reclaim, n (%)			147 (63.9)	56 (62.9)	91 (64.5)	0.80
Cow breed, n (%)		Charolaise	34 (14.8)	0 (0.0)	34 (24.1)	<0.0001
		Crossbreed	141 (61.3)	68 (76.4)	73 (51.8)	
		Limousine	55 (23.9)	21 (23.6)	34 (24.1)	
Bull breed, n (%)		Charolaise	57 (24.8)	7 (7.9)	50 (35.5)	<0.0001
		Crossbreed	15 (6.5)	15 (16.9)	0 (0.0)	
		Limousine	158 (68.7)	67 (75.3)	91 (64.5)	
Cow origin, n (%)		France	149 (64.8)	48 (53.9)	101 (71.6)	0.006
		Italy	81 (35.2)	41 (46.1)	40 (28.4)	
Bull origin, n (%)		France	145 (63.0)	46 (51.7)	99 (70.2)	0.005
		Italy	85 (37.0)	43 (48.3)	42 (29.8)	
Concern of farmers regarding foot diseases^$^, n (%)		1	23 (10.0)	7 (7.9)	16 (11.4)	0.50
		2	26 (11.3)	10 (11.2)	16 (11.4)	
		3	46 (20.0)	15 (16.9)	31 (22.0)	
		4	62 (27.0)	23 (25.8)	39 (27.7)	
		5	73 (31.7)	34 (38.2)	39 (27.7)	

Data refer to the last 5 years (2016–2021). (*) Pasture; (**) Pasture + hay; (***) Pasture + hay + concentrates only in critical periods (lactation, breeding); (****) Pasture + hay + concentrates all days.

($) The level of concern is indicated by a bracket from 1 to 5, where 1 represents the lowest degree of concern and 5 represents the highest.

The Charolaise cow and bull breed were more prevalent in farms with lameness recurrence in comparison with the cross and Limousine breeds. Concern on foot diseases was highest in farmers with herds with lameness issues (p<0.0001). Farmers who performed treatments had a low prevalence of lameness issues, in comparison with role played by podiatrists and veterinarians. French cows did not show lameness issues (p = 0.006) in comparison with Italian cows.

Farmers who performed treatments had a lower level of concern (50.0%) than podiatrists and veterinarians (18.8% and 31.3%, respectively) (Table 2).

**Table 2:** Personnel who performed the treatments and concern of farmers regarding foot diseases.

		Concern of farmers regarding foot diseases^$^					p-value
		1	2	3	4	5	
Personnel who performed the treatments, n (%)	Farmer	8 (50.0)	5 (31.3)	4 (12.9)	6 (15.4)	8 (20.5)	0.007
	Podiatrist	3 (18.8)	3 (18.8)	14 (45.2)	8 (20.5)	4 (10.3)	
	Veterinary	5 (31.3)	8 (50.0)	13 (41.9)	25 (64.1)	27 (69.2)	

($) The level of concern is indicated by a bracket from 1 to 5, where 1 represents the lowest degree of concern and 5 represents the highest.

Farmers who performed treatments had a lower level of satisfaction (50.0%) than podiatrists and veterinarians (18.8% and 31.3%, respectively).

Pure cow breed and French bull origin were the only two farm characteristics showing association with lameness issues. Farms with pure cow breeds had 2.37 times higher odds of having lameness issues when compared with farms without pure cow breeds (OR = 2.37, 95% CI = 1.24–4.52, p = 0.009), whereas farms with French bull origin had 1.97 times higher odds of having lameness issues in comparison with farms without French bull origin (OR = 1.97, 95% CI = 1.11–3.51, p = 0.02). Farmer’s age was a significant predictor of lameness (OR = 0.98, 95% CI: 0.96, 1.00, p = 0.02) after controlling for other variables, indicating that for each one-year increase of the variable age, the odds of developing lameness decreased by 2%.

## Discussion

Knowledge on foot diseases in the cow-calf line is of paramount importance for the farm management. In fact, beef cattle in extensive farming systems should achieve long distances to feed adequately [22,23].

In agreement with other studies, a strong association was found between breed (of both bulls and cows) and the occurrence of diseases and recurrences [24]. This finding is very relevant since natural mating is the only system used in 228 of the 230 (99.1%) farms. Thus, the choice of bull seems to be fundamental for the transmission of useful traits: a high incidence of animals with foot diseases was found in the Charolais breed, (100% of farms with Charolais had foot diseases in cows and 87.7% in bulls) and an equal incidence of recurrences (94% of cows and 68% of bulls). Bulls purchased from a foreign country, especially those from France, seem to be more susceptible to the occurrence of foot diseases, whereas in female cows, the country of origin appears to be associated with a higher incidence of both disease and its recurrence (p< 0.006). This finding shows how it could be key to genetically select animals facing extensive breeding and rougher environments. As shown in Table 3, pure cow breed and French bull origin are two important farm characteristics associated with lameness issues in cows. The finding that farms with pure cow breeds had higher odds of lameness issues is consistent with previous studies focused on intensive farming (purebred cows are more susceptible to lameness due to genetic factors) [25]. Farmers with purebred cows should be more vigilant in monitoring their cows for lameness issues and take proactive measures to prevent or manage risk factors. Similarly, the finding that farms with French bull origin had higher odds of lameness issues highlights the importance of considering the origin of bulls used for breeding in the selection of cows. A study by Nieuwhof and Bishop (2005) found that lameness was higher in farms using bulls from North America than farms using bulls from Europe [26]. In terms of farm management, several studies showed nutrition and farm management as predisposing factors [27,28]. As grazing is the predominant source of feed for these herds, no association was found between feed ration and disease incidence and recurrence (Table 4). Hay and other feed supplements constitute a minor percentage of the diet, which explains why podalic disease caused by acidosis from excessive carbohydrates and lameness produced by a high level of ammonia from excessive protein in the ration are uncommon [29,30]. The association between soil type and management and the occurrence and recurrence of disease is another specific feature of farm management [31–34]. In our geographical area, the soil has a relatively heterogeneous texture; in the mountainous region, the soil is predominantly rocky, with a high percentage of granite, whereas in the coastal regions, the soil is predominantly sandy. This inhomogeneity help evaluate the role played by different soil textures on animals who spend 99 percent of their lives outdoors on pastures before being fattening and slaughter. The soil had a little effect on the occurrence of podalic diseases and recurrences. Soil can affect the incidence of lameness in grazing animals because the growth of microorganisms responsible for lameness is influenced by the precipitation [35,36]. The discrepancy between the scientific evidence and our study can be explained by poor precipitations during the study period [37]. Moreover, the spread of microrganisms between animals is limited because of abundant grazing space, resulting in a lower population density [38,39].

**Table 3:** Logistic regression analysis to assess the relationship between farm characteristics and lameness issues.

		Univariate analysis		Multivariate analysis	
		OR (95% CI)	p-value	OR (95% CI)	p-value
Farmer’s age, years		0.98 (0.96–0.99)	0.02	0.98 (0.96–1.00)	0.02
Farm’s age, years		1.00 (0.98–1.01)	0.77	-	-
No. of animals per farm		0.99 (0.98–1.00)	0.21	-	-
No. of animals per farm	0–30	Ref.	Ref.	Ref.	Ref.
	31–60	1.31 (0.51–3.37)	0.58	-	-
	61–90	1.09 (0.47–2.53)	0.84	-	-
	91–120	0.78 (0.32–1.92)	0.59	-	-
	>120	0.47 (0.09–2.50)	0.38	-	-
Full-time activity		1.05 (0.55–2.01)	0.89	-	-
Diet	P*	Ref.	Ref.	Ref.	Ref.
	P+H**	1.02 (0.49–2.15)	0.96	-	-
	P+H+CC***	1.24 (0.60–2.56)	0.56	-	-
	P+H+CA***	1.27 (0.37–4.38)	0.71	-	-
Rocky soil ≥50%		0.60 (0.25–1.42)	0.24	-	-
Land reclaim		1.07 (0.62–1.86)	0.80	-	-
Pure cow breed		3.01 (1.67–5.44)	<0.0001	2.37 (1.24–4.52)	0.009
French cow origin		2.16 (1.24–3.76)	0.007	1.67 (0.90–3.09)	0.10
French bull origin		2.20 (1.27–3.82)	0.005	1.97 (1.11–3.51)	0.02
Concern of farmers regarding foot diseases^$^, n (%)	1	Ref.	Ref.	Ref.	Ref.
	2	0.70 (0.21–2.30)	0.56	-	-
	3	0.90 (0.31–2.67)	0.86	-	-
	4	0.74 (0.27–2.07)	0.57	-	-
	5	0.50 (0.19–1.36)	0.18	-	-

**Table 4:** Farms characteristics stratified between absence or presence of lameness recurrence in the farms.

		Number of farms without recurrence (n = 69)	Number of farms with recurrence (n = 72)	p-value
Diet, n (%)	P***	12 (17.4)	15 (20.8)	0.72
	P+H***	24 (34.8)	21 (29.1)	
	P+H+CC***	30 (43.5)	30 (41.7)	
	P+H+CA***	3 (4.4)	6 (8.3)	
Soil, n (%)	Rocky (R)	20 (29.0)	25 (34.7)	0.80
	Sandy (S)	1 (1.5)	1 (1.4)	
	50% R-50% S	24 (34.8)	23 (31.9)	
	75% R-25%S	13 (18.8)	16 (22.2)	
	75% S-25% R	11 (15.9)	7 (9.7)	
Land reclaim, n (%)		40 (58.0)	51 (70.8)	0.11
No. of animals per farm, n (%)	0–30	11 (15.9)	8 (11.1)	0.20
	31–60	12 (17.4)	19 (26.4)	
	61–90	25 (36.2)	32 (44.4)	
	91–120	20 (29.0)	11 (15.3)	
	>120	1 (1.5)	2 (2.8)	
Cow breed, n (%)	Charolaise	2 (2.9)	32 (44.4)	<0.0001
	Crossbreed	47 (68.2)	26 (36.1)	
	Limousine	20 (29.0)	14 (19.4)	
Bull breed, n (%)	Charolaise	16 (23.2)	34 (47.2)	0.003
	Crossbreed	-	-	
	Limousine	53 (76.8)	38 (52.8)	
Cow origin, n (%)	France	42 (60.9)	59 (81.9)	0.006
	Italy	27 (39.1)	13 (18.1)	
Bull origin, n (%)	France	47 (68.1)	52 (72.2)	0.59
	Italy	22 (31.9)	20 (27.8)	
Concern of farmers regarding foot diseases^$^, n (%)	1	3 (4.1)	13 (18.1)	<0.0001
	2	1 (1.5)	15 (20.8)	
	3	14 (20.3)	17 (23.6)	
	4	24 (34.8)	15 (20.8)	
	5	27 (39.1)	12 (16.7)	
Personnel who performed the treatments, n (%)	Farmer	3 (4.4)	28 (38.9)	<0.0001
	Podiatrist	9 (13.0)	23 (31.9)	
	Veterinary	57 (82.6)	21 (29.2)	

Data refers to the last 5 years (2016–2021). (*) Pasture; (**) Pasture + hay; (***) Pasture + hay + concentrates only in critical periods (lactation, breeding); (****) Pasture + hay + concentrates all days.

($) The level of concern is indicated by a bracket from 1 to 5, where 1 represents the lowest degree of concern and 5 represents the highest.

Farmers’ experience and knowledge can play a crucial role in preventing lameness issues in agreement with previous studies [40].

**Fig 1:**
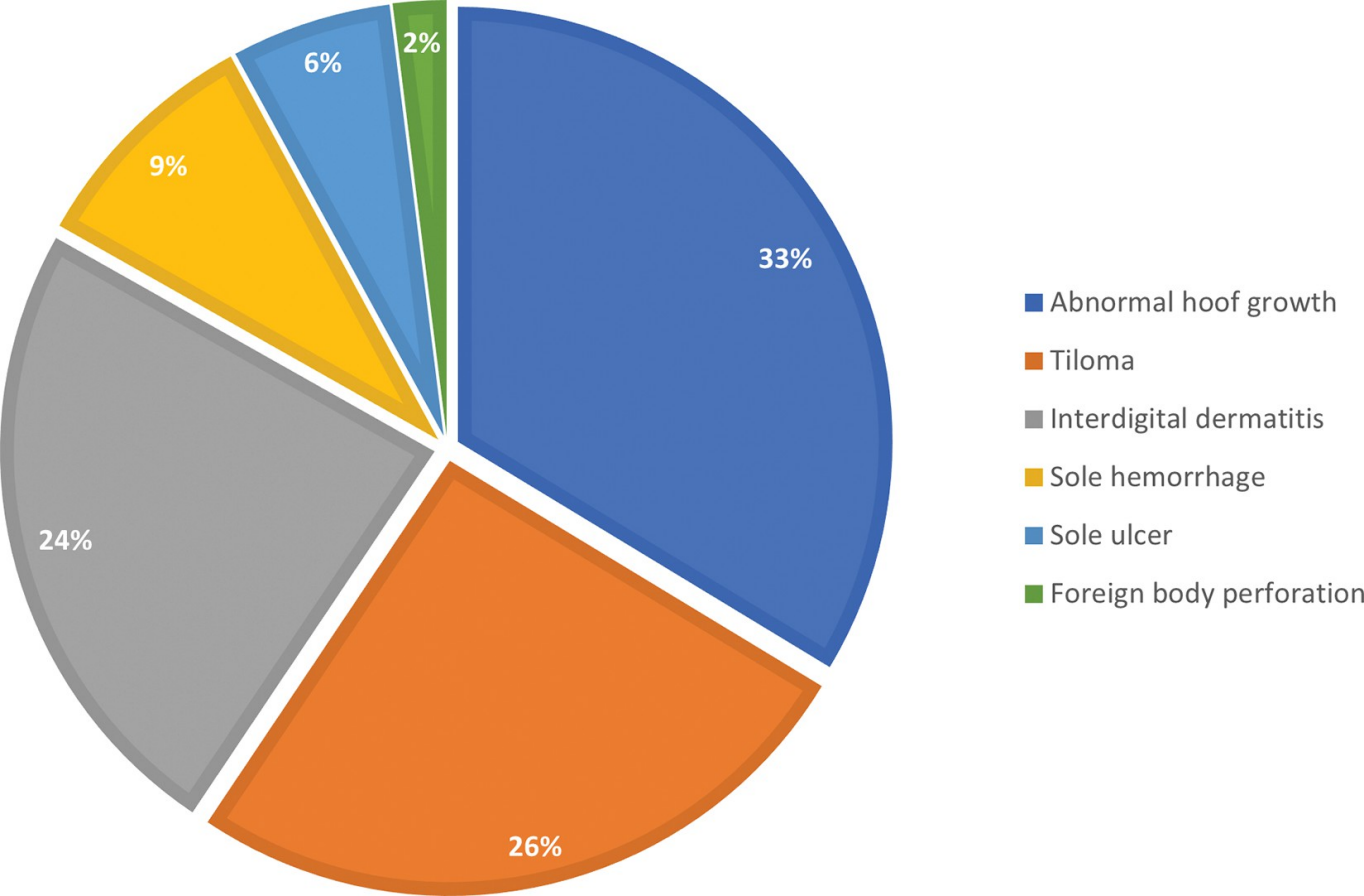
Pie chart of the prevalence of foot diseases. The calculated percentage frequencies were based on the total number of detected pathologies.

As can be seen in Fig 1, the most prevalent clinical conditions are non-infectious, according to hoof disorders in dairy cattle under field conditions [41]. Excluding rare cases of foreign-body perforation of an accidental nature (2%), the most frequent pathologies are abnormal claw growth and tiloma, which account for 60% of the total.

Our study found that herds with farmers who have a low concern for foot health have a significantly higher incidence of recurrences. The choice of professionals who performs the therapy is linked to the farmer’s sensitivity, with therapies performed by a veterinarian being more effective and causing fewer recurrences than those performed by breeders or podiatrists. Satisfaction levels were also higher following therapies performed by a veterinarian (Table 5). These findings emphasize the importance of the professional figure in foot health and highlight the economic losses incurred by farmers due to foot pathologies [42]. The presence of these diseases generates an average weight loss per animal of 90 kg, with the farmer estimating an economic loss of 105 euros per animal. Considering an average number of animals of 63 with a prevalence of 60.87%, the farmers estimated an average annual farm loss of 4026,55€. These results are slightly lower, but consistent with the scientific evidence. The cost per lameness case in cattle was recently estimated in the United States using dynamic programming to be $177.62, ranging from $120.70 to $216.07 per case depending on the specific type of lameness [43]. Veterinarians seem to play a pivotal role in diagnosing and choosing the appropriate therapy, which can have a significant impact on the economic sustainability of the farm.

**Table 5:** Personnel who performed the treatments and level of satisfaction with the treatment.

		Farmer’s level of satisfaction with the treatment^§^					p-value
		1	2	3	4	5	
Personnel who performed the treatments, n (%)	Farmer	8 (50.0)	5 (31.3)	4 (12.9)	6 (15.4)	8 (20.5)	0.007
	Podiatrist	3 (18.8)	3 (18.8)	14 (45.2)	8 (20.5)	4 (10.3)	
	Veterinary	5 (31.3)	8 (50.0)	13 (41.9)	25 (64.1)	27 (69.2)	

(§) The level of satisfaction is indicated by a bracket from 1 to 5, where 1 represents the lowest degree of satisfaction and 5 represents the highest degree of satisfaction.

## Conclusion

This study emphasizes the importance of knowledge regarding foot diseases in the cow-calf line for the management of extensive farming systems. The study found a strong association between breed, country of origin of animals, and lameness issues in cows. Farmers should genetically select animals facing extensive breeding and rougher environments and evaluate the origin of bulls used for breeding when selecting cows to avoid lameness issues. The study also found that the farmer’s age and experience could play a crucial role in preventing lameness issues. No association was found between feed ration and disease incidence and recurrence. Furthermore, poor effect of solil on podalic diseases and recurrences was found. Continuous research and monitoring of cow-calf line management and health is needed to improve animal welfare, productivity, and profitability.

## Supporting information

S1 File (DOCX)

S2 File (XLSX)
