# Supplementary material for: Retrospective study of lameness in beef cattle in northeastern Sardinia, Italy
Source: PLoS One. 2023 May 23;18(5):e0285840. doi: 10.1371/journal.pone.0285840 (PMC10204999; doi:10.1371/journal.pone.0285840)
Supplement: S1 File — (DOCX) [file pone.0285840.s001.docx]

**Survey on foot diseases in beef cattle: Sardinia**

Department of Veterinary Medicine, Via Vienna 2, 07100, SS

**Contact**:

Head researcher: Dr. Sarah Morrone DVM; s.morrone@phd.uniss.it

Supervising professor: Prof. Nicolò Columbano, DVM, PhD.; [ncolumbano@uniss.it](mailto:ncolumbano@uniss.it)

The University of Sassari's Department of Veterinary Medicine is conducting a study on podalic pathologies on beef cattle farms in Sardinia, with a focus on the Gallura region. This research begins with an investigation on the real situation in farms.

For this reason, we would like to request that you complete this survey in which you will be asked to provide some information about your farm, with a focus on the bibliography-referenced predisposing factors for lameness.

Please answer the questions to the best of your knowledge and with as much detail as possible.

All of the information provided by you will be treated as confidential and will only be used for research purposes.

Completed questionnaires can be sent back electronically to [s.morrone@phd.uniss.it](mailto:s.morrone@phd.uniss.it) or in physical form to: *Prof. Nicolò Columbano, Università degli Studi di Sassari - Dipartimento di Medicina Veterinaria,* sezione di Chirurgia, *Via Vienna, 2, 07100 Sassari SS.*

If you have any questions regarding the survey, please don’t hesitate to contact us.

On behalf of the research team we thank you for your participation.


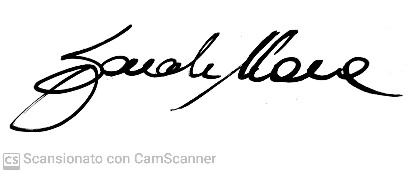


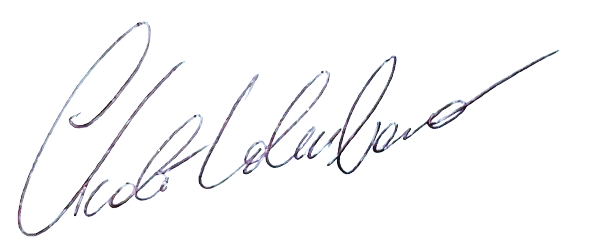
_________________________

Sarah Morrone

__________________________

Nicolò Columbano

Section one: Owner general informations.

1. Title

__________________________

1. Name

__________________________

1. Second name

__________________________

1. Surname

__________________________

1. Home address

__________________________

1. CAP

__________________________

1. City

__________________________

1. Region

__________________________

1. Country

__________________________

1. Date of Birth

__________________________

1. Birthplace

___________________________

1. Qualification

- Primary school
- Middle school
- High school
- Bachelor’s degree
- Master’s degree
- Doctoral degree
- Other

________________________

1. What year was the farm opened?

____________________

1. When did you start working in this farm?

____________________

1. Did the farm previously belong to a family member?

- Yes
- No

1. Is your farm work part-time or full-time?

- Part-time
- Full-time

1. How many hours a week do you spend on the farm?

______________________

Section two: Farm general informations - management

1. Farm address

___________________________

1. Farm code

___________________________

1. Choose the correct farm system from those described below

- Intensive farming system
- Semi-intensive farming system
- Semi-extensive farming system
- Extensive farming system
- Other

___________________

1. Does the farm have at least one water basin?

____________________

1. Has the land been reclaimed?

_____________________

1. Are the animals exclusively fed grass?

- Yes
- No

1. If you answered "no" to the preceding question, please specify what type of supplemental food you feed the animals.

- Hay
- Other supplements

1. If you answered "other supplements" to the previous question, please indicate when these supplements are administered to your animals.

• Only in critical periods (eg.: breeding)

• All days

1. Please specify the soil texture type on the farm.

Rocky

Sandy

50R;50S

75R;25S

25S;75R

Other

______________________

1. What reproductive strategy is employed on the farm?

- AI
- Natural insemination (bulls)
- Both
- Other

_________________________

Section three: Animals - genetic

1. Number of animals in your farm

_____________________

1. Farm-raised breed – BULLS (more than one answer is possible)

- Limousine
- Chairolais
- Simmenthal
- Sarda
- Crossbreed
- Other

__________________

1. Farm-raised breed – COWS (more than one answer is possible)

- Limousine
- Chairolais
- Simmenthal
- Sarda
- Crossbreed
- Other

__________________

1. Indicate the countries of origin of the BULLS

- Italy
- France
- UK
- Germany
- Spain
- The Netherlands
- Other

____________________

1. Indicate the countries of origin of the COWS

- Italy
- France
- UK
- Germany
- Spain
- The Netherlands
- Other

____________________

Section four: Foot diseases

1. How important do you consider foot pathologies in your farm to be on a scale from 1 to 5?
2. Have any changes been observed in the feet of cattle on the farm in the past five years?

- Yes
- No

If you answered "yes" to the previous question, which pathology did you observed?

Multiple responses are possible. BELOW ARE LISTED SOME EXAMPLES TO ASSIST YOU IN INDICATING THE CORRECT ANSWER.

-
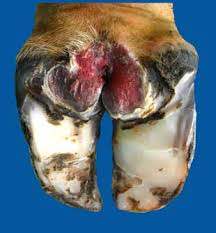
Digital dermatitis
-
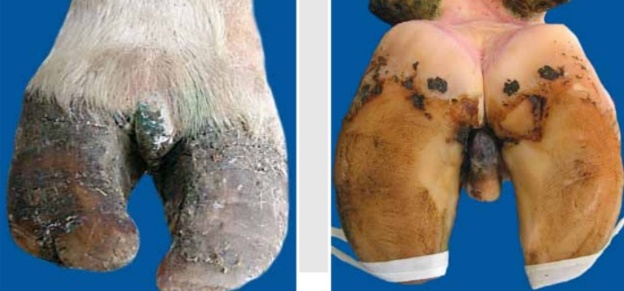
Tiloma


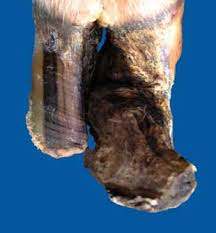


- Overgrowth of the claw


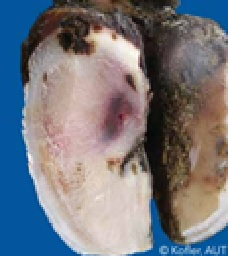


- Sole haemorrhage
- Ulcer of the sole
- Foreign body perforation (e.g. screw)

1. Indicate the estimated amount of weight lost by each animal on the farm due to podalic diseases

____________________________ Kg

1. Indicate the estimated economic loss for each animal on the farm due to podalic disease (including treatment costs)

____________________________ €

Section five: Treatment

1. Have the animals presenting the aforementioned pathologies received treatment?

- Yes
- No

1. Who conducted treatments to animals with podalic diseases?

- Veterinarian
- Podiatrist/maniscalist
- Farmer
- Other

__________________________

1. Please, describe briefly the treatment procedure

- Drugs __________________________________________________________________________________________________________________________________________________________________________________________________________________________________________
- Procedures __________________________________________________________________________________________________________________________________________________________________________________________________________________________________________
- Follow-up indications __________________________________________________________________________________________________________________________________________________________________________________________________________________________________________

1. Did the animals with the disease experience recurrences?

- Yes
- No

1. Please indicate the level of satisfaction with the treatment of diseased animals.

1 2 3 4 5
